# Anomalous transverse resistance in the topological superconductor *β-*Bi_2_Pd

**DOI:** 10.1038/s41467-022-32877-x

**Published:** 2022-09-09

**Authors:** Xiaoying Xu, Yufan Li, C. L. Chien

**Affiliations:** 1 William H. Miller III Department of Physics and Astronomy, Johns Hopkins University, Baltimore, MD 21218 USA; 2 Department of Physics, The Chinese University of Hong Kong, Shatin, Hong Kong; 3 Department of Physics, National Taiwan University, Taipei, 10617 Taiwan

**Keywords:** Superconducting properties and materials, Topological matter

## Abstract

A supercurrent flowing in a superconductor meets no resistance. Yet an electric field may still be established within the superconductor in the presence of dissipative processes, such as vortex motion. Here we report the observation of a transverse voltage drop in superconducting *β-*Bi_2_Pd thin films. Unlike the Hall effect in general or in other superconductors, the sign of the observed transverse voltage does not depend on the external magnetic field. Instead, it is dictated by the broken inversion symmetry on the film interfaces. This anomalous transverse voltage, or transverse resistance, is indicative of a chirality that likely resonates with the topological surface states reported in *β-*Bi_2_Pd.

## Abstract (web summary)

Centrosymmetric *β-*Bi_2_Pd is a candidate topological superconductor. Here, the authors observe a transverse voltage in *β-*Bi_2_Pd thin films and propose that this voltage is a result of chiral topological surface states.

## Introduction

Superconductors are best known as perfect conductors, where electric current flows without resistance, and as perfect diamagnets, with complete expulsion of magnetic flux. However, in the vortex phase of type II superconductors, dissipation may still occur, and allow an electric field to be established within the superconductor. One prominent example is that of the vortex motion manifested as a longitudinal voltage drop, or a resistance^1,2^. It has also been suggested that the vortex motion, especially in high-*T*_c_ superconductors, gives rise to a Hall voltage^2–6^; although the origin of such a Hall effect is yet to be fully understood^7^. In the Hall effect, the transverse voltage must be odd-symmetric to the magnetic field. We investigate, in this study, a transverse voltage drop even-symmetric to the applied field in superconducting *β*-Bi_2_Pd thin films, within the vortex phase to the upper critical field *H*_c2_. The sign of the transverse voltage is dictated not by the polarity of the magnetic field, but by the broken inversion symmetry at the interfaces. This key feature distinguishes the observed transverse resistance from that of the Hall effect, and indicates a chirality likely associated with the topological surface states reported by angle-resolved photoemission spectroscopy (ARPES)^8^ and scanning tunneling microscopy (STM) studies^9,10^.

Superconducting *β-*Bi_2_Pd, a compound with centrosymmetric tetragonal crystal structure, is a candidate for topological superconductors^8–10^. Spin-polarized surface states have been revealed by ARPES to reside on the cleaved (001) plane of bulk crystals^8^, as was further confirmed by an STM study^10^. Signatures of Majorana bound states have also been reported at the center of the vertices in epitaxial thin films, as well as a surface superconducting gap that coexists with the bulk gap^9^. The verdict remains controversial as other tunneling spectroscopy and calorimetric experiments on bulk specimens conclude instead conventional *s*-wave superconductivity^11–13^. In efforts parallel to this work, we have examined the fluxoid quantization in *β-*Bi_2_Pd thin films. Half-quantum fluxoid has very recently been observed in mesoscopic ring devices of *β-*Bi_2_Pd, that evidences unconventional superconductivity^14^. It is highly desirable that the features more directly related to the topological properties of the surface/interface are examined. In the presence of the topological surface states (TSS), one expects that the chiral spin texture of the TSS may introduce novel phenomena in the form of electrical transport properties, when the TSS is placed in the proximity of a magnetic entity. As an example, exotic magnetoresistance has been reported in ferromagnet/topological insulator heterostructures^15,16^. In this study, we report the observation of anomalous transverse resistance in *β-*Bi_2_Pd thin film induced by modified interfaces. We show that it is an interfacial effect with the sign and the amplitude determined by the manner that the interfaces are modified, and discuss the implication of the observed effect with respect to topological surface states.

## Results

**Fig. 1:**
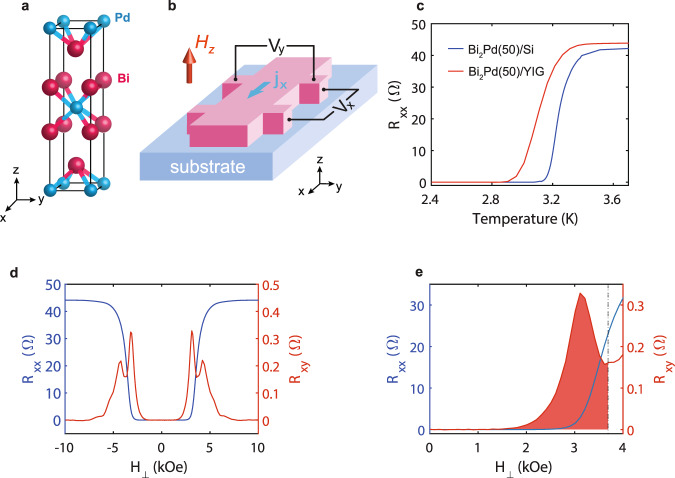
The physical properties of *β-*Bi_2_Pd film. **a** Crystal structure of superconductor *β-*Bi_2_Pd. **b** Experimental setup. The d.c. current was applied in the film plane along the x-axis. Voltage along x- and y-axis were recorded simultaneously. The longitudinal (*R*_xx_) and transverse resistance (*R*_xy_) were derived accordingly. **c** *R*_xx_ as a function of temperature for 50 nm *β-*Bi_2_Pd films which were deposited on oxidized Si and YIG substrates without applied magnetic field. **d** *R*_xx_ (blue line) and *R*_xy_ (red line) versus magnetic field for 50 nm Bi_2_Pd/YIG. **e** Zoomed-in details of **d** The magnetic field is applied perpendicular to the film plane. The dot-dashed line corresponds to the critical field (H_*c*2_).

We use magnetron sputtering to deposit (001)-textured *β-*Bi_2_Pd thin films on polycrystalline ferrimagnetic insulator Y_3_Fe_5_O_12_ (yttrium iron garnet, or YIG) and also on thermally oxidized-silicon substrates. The (001)-textured *β-*Bi_2_Pd thin films, with crystal structure shown in Fig. 1a, have been patterned into Hall bar devices for electrical transport measurement as shown in Fig. 1b, with more details described in the Method section. For the same film thickness of 50 nm, *β-*Bi_2_Pd/YIG has a slightly lower critical temperature (*T*_*c*_ ∼ 3.3 K) than that of *β-*Bi_2_Pd/Si (*T*_*c*_ ∼ 3.4 K), as shown in Fig. 1c. Both are lower than the highest reported *T*_*c*_ of 5.4 K in bulk specimen^17^, but comparable to that of epitaxial thin films (Methods)^18^. We note that the suppression of *T*_*c*_ is common in thin film specimens, due to reduced dimensions.

We first describe the results of the longitudinal (*R*_*xx*_) and transverse (*R*_*xy*_) resistance of the 50 nm-thick *β*-Bi_2_Pd/YIG thin film, with a magnetic field (*H*_⊥_) applied perpendicular to the film plane. When the applied field exceeds the critical field *H*_c2_, the sample becomes non-superconducting, as displayed by the field dependence of *R*_*xx*_ shown in Fig. 1d. We obtain an *H*_c2_ of 3.7 kOe, determined as *R*_xx_ retains 50% of its normal-state value. Whereas, *R*_xy_, on the other hand, manifests a more complex behavior. Within the superconducting phase but below *H*_c2_ (*H*_⊥_ < 1.5 kOe), *R*_xy_ remains zero or negligibly small. When the field is much greater than *H*_c2_ (*H*_⊥_ > 8 kOe), *R*_xy_ displays Hall effect of the normal state, with a Hall coefficient on the order of 1 × 10^−13^ Ω cm/Gauss, however barely visible in the scale as presented in Fig. 1d. Unexpectedly, *R*_*xy*_ shows peaks at the field regions close to *H*_c2_.

**Fig. 2:**
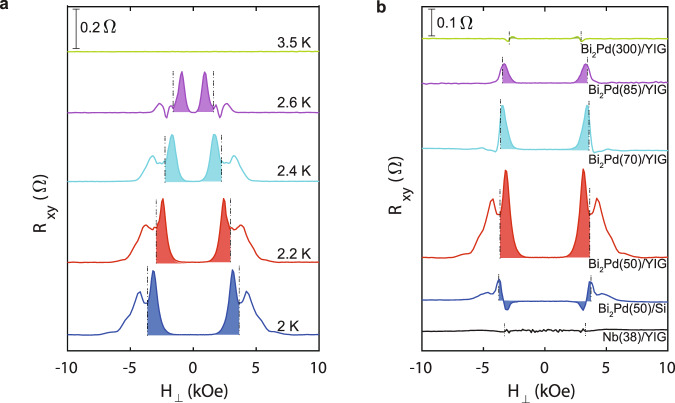
Transverse resistance, *R*_*xy*_, (with arbitrary offsets for clarity) as a function of magnetic field. **a** R_xy_ at various temperatures for 50 nm Bi_2_Pd/YIG film. **b** R_xy_ for Bi_2_Pd/YIG films with different thicknesses (50 nm, 70 nm, 85 nm, 300 nm, respectively) in comparison with 50 nm *β-*Bi_2_Pd/Si and 38 nm Nb/YIG. In order to compare the results obtained from the samples with various *T*_c_, the temperatures at which the measurements are conducted are selected so that *H*_c2_ remains similar values for all the samples. The temperatures are: 7.0 *K*, 2.4 K, 2.0 K, 2.2 K, 2.3 K and 2.6 K for thin films Nb(38)/YIG, Bi_2_Pd(50)/Si, Bi_2_Pd(50)/YIG, Bi_2_Pd(70)/YIG, Bi_2_Pd(85)/YIG and Bi_2_Pd(300)/YIG, respectively. 10 μA d.c. current was applied in-plane. All the magnetic fields are applied perpendicular to the film plane. The dot-dashed lines correspond to the critical fields (H_*c*2_).

The surprise comes in two-fold. First, a non-zero *R*_*xy*_ rises from well within the superconducting phase (*H*_⊥_ ∼ 1.5 kOe), much earlier than *R*_*xx*_ shows any appreciable rise that only occurs when *H*_⊥_ further increases to about 3 kOe. In other words, a transverse electric field has been established when the sample is still essentially superconducting, despite the formation of vortices. Fig. 1e presents an expanded view from zero field to *H*_c2_, where the *R*_*xy*_ anomaly that occurs within the superconducting phase (when *H*_⊥_ < *H*_c2_) is shaded in red. We show further evidences that the *R*_*xy*_ anomaly is related to the superconducting phase and the phase transition at *H*_c2_. This is shown in Fig. 2a for *β-*Bi_2_Pd(50 nm)/YIG at various temperatures. At higher temperatures, the occurrence of the *R*_xy_ anomaly continuously moves to lower fields in accord to the suppression of *H*_c2_, the positions of which are marked by the black dashed lines. The magnitude of the *R*_*xy*_ anomaly is only slightly suppressed at higher temperatures, by about 30% at 2.6 *K,* before it abruptly vanishes above *T*_c_.

The second surprise is that the *R*_*xy*_ anomaly is even-symmetric to the perpendicularly applied magnetic field, i.e., *R*_*xy*_(*H*_⊥_) = *R*_xy_(−*H*_⊥_). This is in sharp contrast to the well-known universal hallmark of all the known Hall effects, that it is always of odd symmetry to the magnetic field, *R*_*xy*_(*H*_⊥_) = − *R*_*xy*_(−*H*_⊥_). We note that the observation of a transverse resistance is effectively an identification of chirality: as the charge carriers travel along the x−axis, they experience an electric field along the y−axis, either pointing to the left or to the right. For the case of the ordinary Hall effect, the chirality is governed by the Lorentz force, $\overrightarrow{{{{{{{{\bf{F}}}}}}}}}\,=\,\overrightarrow{{{{{{{{\bf{j}}}}}}}}}\times \overrightarrow{{{{{{{{\bf{B}}}}}}}}}$. It hence brings a more profound symmetry issue to the observed even-symmetric *R*_xy_: what, if not the magnetic field, determines the polarization of the transverse electric field? The centrosymmetric crystalline structure of *β-*Bi_2_Pd and the polycrystalline nature of the thin film, forbid any bulk-originated chirality. On the other hand, chirality may also originate from the breaking of the inversion symmetry on the interfaces. The chiral spin-momentum locking of the topological surface states is a well-known example. By measuring several *β-*Bi_2_Pd(*t*)/YIG samples with different *β-*Bi_2_Pd thickness *t*, we have indeed found evidence that the *R*_*xy*_ anomaly originates from the *β-*Bi_2_Pd-YIG interface. The results of a series of *β-*Bi_2_Pd(*t*)/YIG samples with *t* = 50, 70, 85, and 300 nm are shown in Fig. 2b. With increasing *β-*Bi_2_Pd film thickness, the *R*_*xy*_ anomaly steadily decreases in magnitude. When the film thickness reaches 300 nm, the *R*_*xy*_ anomaly is barely visible. These results conclusively demonstrate that the *R*_*xy*_ anomaly is an interfacial effect, which diminishes by increasing the film thickness. The *R*_*xy*_ anomaly, also intimately related to the interface with the ferromagnetic insulator YIG, is dramatically suppressed when the YIG substrate is replaced by oxidized silicon, as shown in Fig. 2b. Finally, no such effect has been observed in the superconducting Nb/YIG heterostructure, as also shown in Fig. 2b, indicating that it is a unique property of superconducting *β-*Bi_2_Pd, unattainable in isotropic *s-*wave superconductors.

**Fig. 3:**
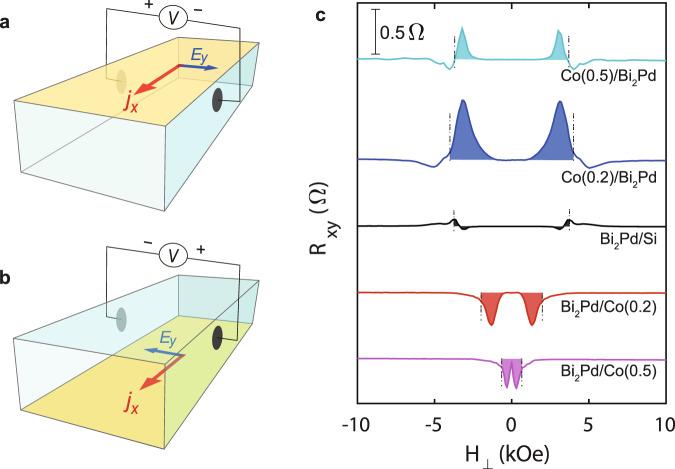
Transverse resistance with cobalt layer under or on top of 50 nm *β-*Bi_2_Pd thin films. The schematic drawings illustrate the origin of transverse resistance (**a**) when cobalt layer is on top of and **b** when cobalt layer is under Bi_2_Pd film. **c** Transverse resistance (with arbitrary offsets for clarity) as a function of the magnetic field of 50 nm-thick Bi_2_Pd films with top or bottom interfaces modified by cobalt layer. The experimental data presented are: Bi_2_Pd/Co (0.5 nm)/Si at 1.7 *K*; Bi_2_Pd/Co (0.2 nm)/Si at 2.0 K; pristine Bi_2_Pd/Si at 2.4 K; Co (0.2 nm)/Bi_2_Pd/Si at 2.3 K; Co (0.5 nm)/Bi_2_Pd/Si at 1.8 K. All the magnetic fields are applied perpendicular to the film plane. The dot-dashed lines correspond to the critical fields (*H*_*c*2_).

Suppose that the *β-*Bi_2_Pd-YIG interface is embedded with a particular chirality that gives rise to the even-symmetric *R*_xy_, the effect is experimentally observable because *β-*Bi_2_Pd is in contact with magnetic YIG on only one interface. Otherwise, imagining performing the experiment on a YIG/*β-*Bi_2_Pd/YIG sandwich structure, an equally chiral top interface would have produced a transverse voltage of the same magnitude but of the opposite sign as that of the bottom interface, as depicted in Fig. 3a, b. Therefore the total *V*_xy_ would have been zero. Equivalently, one may test the conjecture by depositing a YIG layer on top of *β-*Bi_2_Pd/Si, and expect to observe an equally pronounced but opposite *R*_*xy*_. Unfortunately, depositing a YIG top layer is formidably difficult, as the high temperature required to synthesis YIG film would irreparably damage the *β-*Bi_2_Pd layer. Instead, we use a few monolayers of Co to interface either the top or the bottom surface of *β-*Bi_2_Pd. As shown in Fig. 3c, a 0.2 nm ∼ 0.5 nm-thick Co layer is deposited before or after, i.e., situated below or above, the 50 nm-thick *β-*Bi_2_Pd layer. The proximity to a Co layer markedly suppresses *T*_c_, while the effect is more prominent if the Co layer is deposited prior to the growth of the *β-*Bi_2_Pd layer. As a result, *H*_c2_ of the *β-*Bi_2_Pd/Co/Si samples is remarkably lower. Nevertheless, both *β-*Bi_2_Pd/Co/Si and Co/*β-*Bi_2_Pd/Si samples display a pronounced *R*_*xy*_, while the pristine *β-*Bi_2_Pd/Si thin film do not. Most importantly, the sign of *R*_*xy*_ depends on precisely whether Co is placed on the top interface or the bottom interface.

## Discussion

Now we discuss the implications of the experimental observations and the possible mechanism for the even-symmetric *R*_*xy*_ anomaly. In the preceding paragraphs we have elaborated on the conspicuous difference between the *R*_*xy*_ anomaly in *β-*Bi_2_Pd and the Hall effect, that they manifest different symmetries to the magnetic field. Therefore the even-symmetric *R*_*xy*_ must not share the same origin as the Hall resistance reported in some *s*−wave superconductors^2,3^ and high-*T*_c_ superconductors^4–6^. One can also rule out the transverse resistance due to anisotropic resistivity, which are the off-diagonal elements of the resistivity tensor rooted from the symmetry of the crystalline symmetry. Such a contribution, if any, could be expected for single crystals but averaged out in polycrystals. Spontaneous transverse voltage has also been observed in high-*T*_c_ cuprates as a result of electronic nematicity^19^. Again, the nematic director has a set orientation with respect to the crystal axes; therefore the effect shall not be expected in polycrystalline specimen. On the other hand, even-in-field transverse voltage has been observed in a number of superconductors at close vicinity to the superconductor-to-normal-state phase transition, either driven by the temperature^20–22^ or the magnetic field^23^. Such an effect is usually difficult to reproduce and therefore hints of spurious origin^21,23^. It has been proposed to have resulted from slight inhomogeneity within the sample^23^. This effect differs from the *R*_*xy*_ anomaly we observed in *β-*Bi_2_Pd in two major aspects: (i) its magnitude and sign are subject to arbitrary inhomogeneity in each individual sample, in stark contrast to our core findings that the observed *R*_*xy*_ anomaly can be manipulated by modifying interfaces of *β-*Bi_2_Pd. (ii) the onset of the inhomogeneity induced transverse voltage strictly follows that of the longitudinal resistance; its key signature is the proportionality to ∂*V*_xx_/∂*H*^23^. This is, however, not the case for the even-symmetric *R*_*xy*_ that we observed (See supplementary information). We conclude that our experimental findings cannot be explained by random inhomogeneity of the samples, but signify unique properties of the *β-*Bi_2_Pd thin films.

Asymmetric pinning is known to give rise to the guided vortex motion^24–26^ as well as the ratchet effect^27–29^. Under an applied d.c. electric current, the guided motion of vortices may manifest an even-in-field *R*_*xy*_^26^. The orientational asymmetric pinning, key to realizing such an effect, is usually achieved by fabricating particular lithographic patterns onto the superconducting films. The shapes or magnetic properties of the patterns, which also have to be asymmetric, are elaborately designed so that to define the direction of the guided motion^24,26–29^. We note that an interesting analog may be made comparing to our experimental findings. In our case, a modified interface of *β-*Bi_2_Pd appears to take the place of artificial patterning in terms of providing the broken symmetry which gives rise to the even-symmetric *R*_*xy*_. This is to suggest that, instead of orientational pinning sites by design, the presumed topological surface states of *β-*Bi_2_Pd may introduce the corresponding chirality that determines the sign and the magnitude of the effect. We should point out, however, that despite the apparent relevance, it is less clear if guided vortex motion is present and responsible for the even-symmetric *R*_*xy*_ in our system. As we apply the magnetic field in the film plane and parallel to current direction, an even-symmetric *R*_*xy*_ is still observed, with the same sign as in the out-of-plane-field configuration, albeit reduced in magnitude. This indicates that the role played by the magnetic field is to drive the system to the vicinity of the superconducting-normal-state transition. What then is the dissipative mechanism responsible for the transverse voltage in the superconducting phase remains to be explored. To conclude, we observed even-in-field *R*_*xy*_ an anomaly that develops from the superconducting phase of *β-*Bi_2_Pd to the superconductor-to-normal-state transition. We demonstrate the *R*_xy_ anomaly originates from the interface of superconducting *β-*Bi_2_Pd that is in proximity to a magnetic substrate or adatoms. The topology of such a heterostructure dictates the sign of the transverse electric field. These features suggest that this novel phenomenon may originate from the topological surface state of *β-*Bi_2_Pd.

## Methods

### *β-*Bi_2_Pd thin film growth

*β-*Bi_2_Pd were deposited by d.c. magnetron sputtering on heated substrates at 400 °C. (001)-textured films were grown on YIG and thermally oxidized-silicon substrates placed side-by-side. X-ray diffraction of *β-*Bi_2_Pd/YIG and *β-*Bi_2_Pd/Si thin films shows only the *β-*Bi_2_Pd phase, with exclusively (001) texture as shown in Supplementary Fig. 1a (See supplementary information). Both the *β-*Bi_2_Pd/YIG and *β-*Bi_2_Pd/Si films have no in-plane directional preference. The residual-resistivity ratio (RRR) between the resistivity at room temperature and that at 4 *K* of 50 nm *β-*Bi_2_Pd/YIG is 1.55, which is close to that of the epitaxial film grown by molecular beam epitaxy (MBE)^18^.

### Transport measurements

All as-grown film samples were capped with 1 nm-thick MgO as protective layers before taken out of the vacuum chamber. For electrical transport experiments, the thin films were patterned into Hall bars (20 μm long, 10 μm wide) by photolithography. 10 μA d.c. current was applied and the longitudinal and transverse voltages were measured simultaneously.

## Supplementary information

Supplementary Information

## Data Availability

The datasets generated in this study have been deposited in the Harvard Dataverse under accession code 10.7910/DVN/K33GIC.
