# Supplementary material for: Anomalous transverse resistance in the topological superconductor β-Bi2Pd
Source: Nat Commun. 2022 Sep 9;13:5321. doi: 10.1038/s41467-022-32877-x (PMC9463149; doi:10.1038/s41467-022-32877-x)
Supplement: Supplementary file 1 — Supplementary Information [file 41467_2022_32877_MOESM1_ESM.pdf]

Supplementary Information for Anomalous transverse resistance in the topological superconductor β–Bi_2Pd

Xiaoying Xu[1,*], Yufan Li[1,2,*], C. L. Chien[1,3,*]

[1] *William H. Miller III Department of Physics and Astronomy, Johns Hopkins University, Baltimore, MD 21218, USA.*

[2] *Department of Physics, The Chinese University of Hong Kong, Shatin, Hong Kong.*

[3] *Department of Physics, National Taiwan University, Taipei, 10617 Taiwan.*[*]

(Dated: September 8, 2022)

β–Bi_2Pd epitaxial thin films

In Fig. 3c, we demonstrated that the sign of the R_{xy} anomaly may be manipulated by adding a top or bottom layer to polycrystalline β–Bi_2Pd deposited on oxidized Si substrate. We have conducted the same experiment using epitaxial β–Bi_2Pd films. The epitaxial films were deposited on MgO(001) and $SrTiO_3$(001) substrates, where β–Bi_2Pd has its in-plane $< 100 >$ directions parallel to MgO $< 110 >$ directions and β–Bi_2Pd $< 100 >$ parallel to $SrTiO_3$ $< 100 >$ directions. A thin layer of Co ($<$ 0.5 nm) may be deposited on the top surface of β–Bi_2Pd, or on the substrate prior to the deposition of β–Bi_2Pd. The epitaxial samples display higher RRR and lower residual resistivity than polycrystalline films. For example, the RRR of 50 nm-thick β–Bi_2Pd/$SrTiO_3$ (001) film reaches ~2.3. In epitaxial samples with improved crystalline quality, the manipulation of the sign of the R_{xy} anomaly is also realized by adding the Co top/bottom layers, as shown in Supplementary Fig. 4 and 5.

Discussions on the trivial explanations of observed anomalous transverse resistance

Transverse resistance has been observed in conventional s–wave superconductors (SCs) and high temperature SCs, although the origin remains debatable [1–4]. A. Segal *et al.* have demonstrated that the transverse resistance with even symmetry to the applied perpendicular magnetic field may originate from the inhomogeneity of the samples [4]. The sign and magnitude of the transverse resistance reflect the inhomogeneity among different samples, thus not possible to manipulate experimentally. It can be demonstrated that the transverse resistance due to inhomogeneity is stringently proportional to $\frac{\partial R_{xx}}{\partial H}$. We have also observed this kind of inhomogeneity-induced transverse resistance in Nb thin films, with three examples shown in Supplementary Fig. 2. The transverse resistance in these Nb films scales closely with $\frac{\partial R_{xx}}{\partial H}$. The signs of transverse resistance may be opposite as shown in the two samples as shown in Supplementary Fig. 2(a) and 2(b). However, the inhomogeneity-induced transverse resistance can be minimized by, for example, placing the substrate near the center of the uniform deposition area. Indeed, we have observed a negligible transverse resistance in such uniform Nb thin films, as shown in Supplementary Fig. 2(c) and Fig. 2b.

The transverse resistance in β–Bi_2Pd thin films show features that are distinctly different from those of the aforementioned inhomogeneity-induced artifact in Nb films. The transverse resistance, always of the same sign, appears in all β–Bi_2Pd/YIG thin films including those with high layer homogeneity. One example is illustrated in Supplementary Fig. 3, obtained

from 50 nm β−Bi_2Pd/YIG at 2.0 K. Evidently the field dependence of R_{xy} shows two peaks, neither of which follows that of $\frac{\partial R_{xx}}{\partial H}$. The left peak of R_{xy} appears at a magnetic field significantly lower than the peak position of $\frac{\partial R_{xx}}{\partial H}$. The R_{xy} anomaly of β−Bi_2Pd, unrelated to inhomogeneity, is possibly due to the inherent chirality of the interfaces. In Fig.3, we show that the sign of the R_{xy} anomaly of β−Bi_2Pd is determined by the interfaces, and the magnitude of R_{xy} decays as the film thickness of β−Bi_2Pd increases, as expected for an interfacial effect, as shown in Fig. 2. These results are entirly different from those attributed to inhomogeneity as reported in conventional supercondcutors.

Angular dependence of R_{xy} on β−Bi_2Pd/YIG in the out-of-plane magnetic field

We patterned the β−Bi_2Pd/YIG thin films along various directions to exclude the extrinsic factors and measured the corresponding R_{xy} in these Hall bars in Supplementary Fig. 6. Since the YIG substrate is polycrystalline, the zero degree of the angle is arbitrary. R_{xy} in various Hall bars remains the same sign and of very similar values.

R_{xy} on β−Bi_2Pd/YIG in the in-plane magnetic field

When the magnetic field is applied in the film plane of β−Bi_2Pd/YIG, either perpendicular [Supplementary Fig. 7 (a)] or parallel [Supplementary Fig. 7 (b)] to the longitudinal electrical current direction, a positive even-symmetric R_{xy} is observed, i.e., with the same sign as the case of out-of-plane field. R_{xy} is smaller in amplitude in the parallel configuration [Supplementary Fig. 7 (b)]. Together with the out-of-plane R_{xy} results discussed in the main text, our experiment establishes that the role of magnetic field is only to push the system towards the superconducting-normal state transition, but not to dictate the sign of R_{xy}. Another broken symmetry must account for the onset of R_{xy}, which invites theoretical investigations.

Magnetization of YIG and Co layers

The M-H curve of the YIG substrate at 2 K shows no hysteresis (Supplementary Fig. 8). The magnetization is saturated at 2 kOe. The M-H curves from 2 K to 5 K show no notable difference. Evidently the field dependence of R_{xy} is unrelated to the magnetization of the YIG substrate. For samples with Co modified interfaces, the Co layer is less than 1 nm in thickness. No magnetization can be detected from the Co layers.

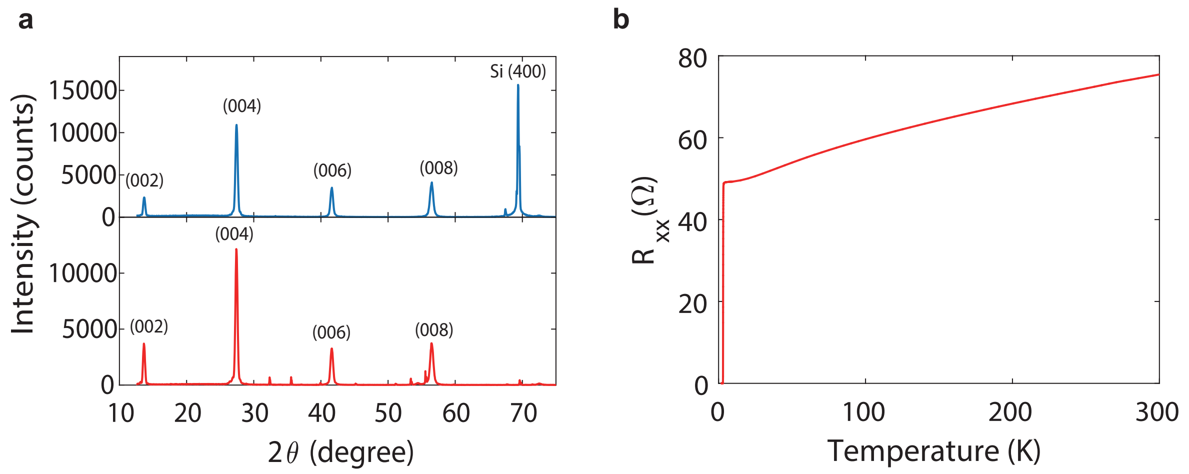

FIG. **Supplementary Fig. 1. The characterization of β-Bi_2Pd film.** (a) x-ray diffraction of 50 nm β-Bi_2Pd films on thermal oxidized Si (top panel) and YIG substrate (lower panel) with (001) texture. (b) Temperature dependence of longitudinal resistance (R_{xx}) of 50 nm β-Bi_2Pd/YIG.

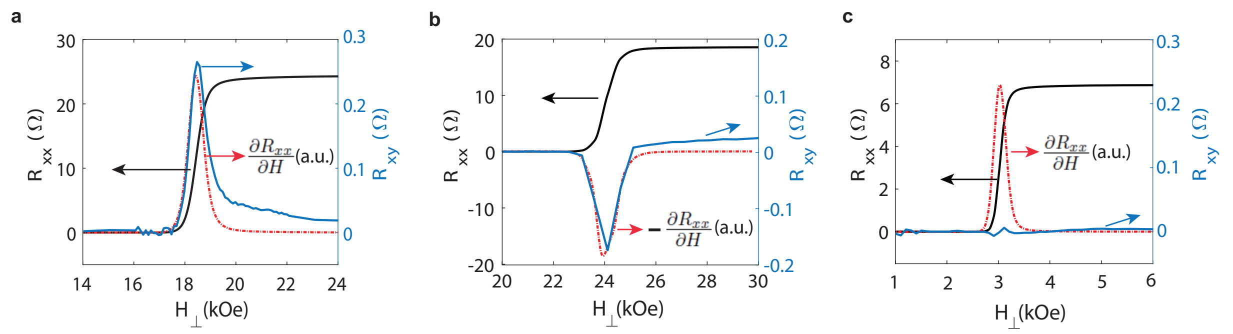

FIG. **Supplementary Fig. 2. Longitudinal R$_{xx}$, transverse resistance R$_{xy}$, and $\frac{\partial R_{xx}}{\partial H}$ near the transition magnetic field in three 38 nm Nb/YIG films.** R$_{xy}$ of the samples in (a) and (b) have opposite signs. R$_{xy}$ of the Nb/YIG film in (c) is negligible.

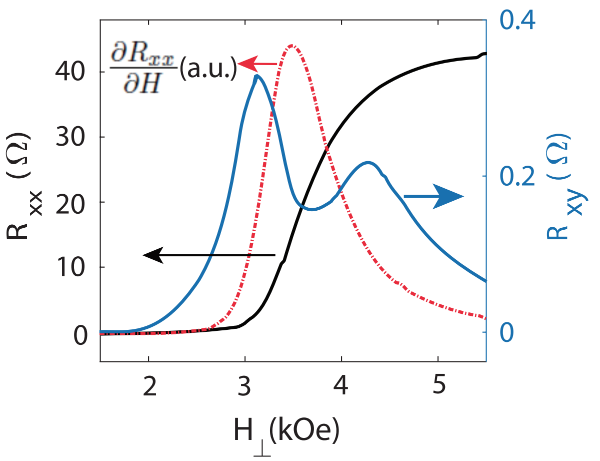

FIG. **Supplementary Fig. 3. Longitudinal R$_{xx}$, transverse resistance R$_{xy}$, and $\frac{\partial R_{xx}}{\partial H}$ near the transition magnetic field in 50 nm β-Bi$_2$Pd/YIG.**

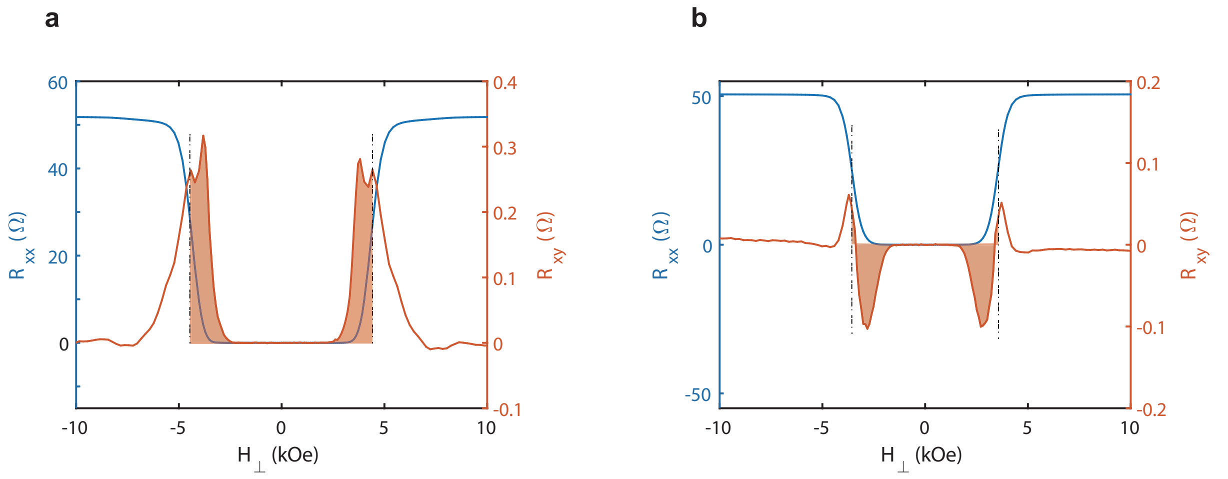

FIG. **Supplementary Fig. 4. (a) Longitudinal (R$_{xx}$) and transverse resistance (R$_{xy}$) in (a) Co(0.5 nm)/β-Bi$_2$Pd(50 nm)/MgO(001) thin film and (b) β-Bi$_2$Pd(50 nm)/Co(0.5 nm)/MgO(001) thin film. Magnetic filed is applied perpendicular to the sample surface.**

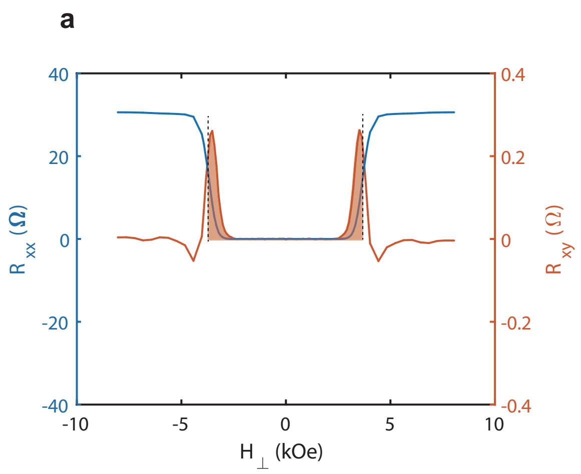

FIG. **Supplementary Fig. 5. (a) Longitudinal (R_{xx}) and transverse resistance (R_{xy}) in (a) Co(0.3 nm)/β-Bi_2Pd(50 nm)/$SrTiO_3$(001) thin film and (b) β-Bi_2Pd(50 nm)/Co(0.4 nm)/$SrTiO_3$(001) thin film. Magnetic filed is applied perpendicular to the sample surface.**

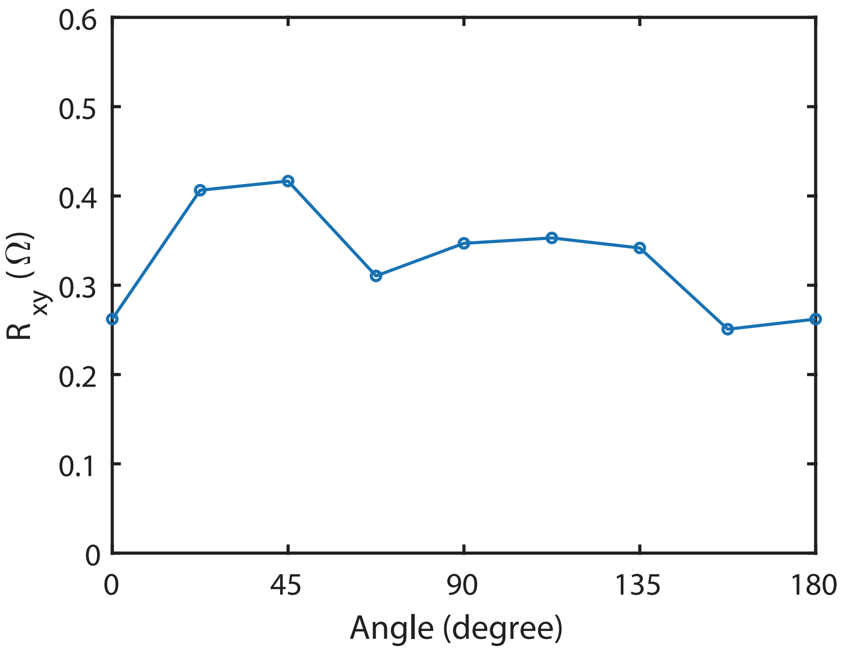

FIG. **Supplementary Fig. 6. Transverse resistance (R_{xy}) in various Hall bars along different direction in β–Bi_2Pd/YIG. The magnetic field is applied out of the sample surface plane.**

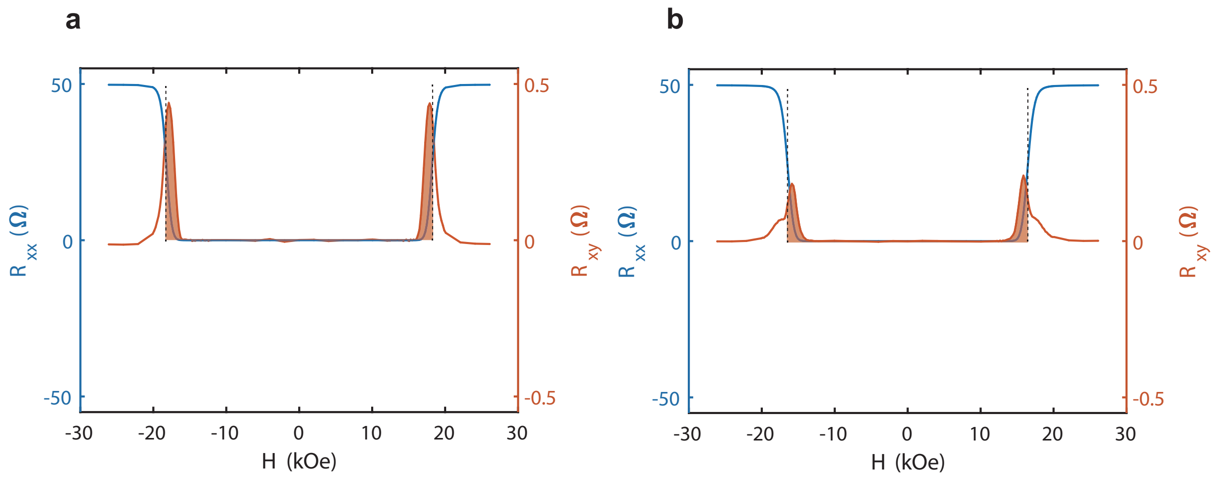

FIG. **Supplementary Fig. 7. (a) Longitudinal ($\mathbf{R}_{xx}$) and transverse resistance ($\mathbf{R}_{xy}$) with magnetic field applied in the surface plane of polycrystalline β-$\mathbf{Bi_2Pd}$(50 nm)/YIG thin film: (a) In-plane magnetic field is perpendicular to electrical d.c. current. (b) In-plane magnetic field is parallel to electrical d.c. current.**

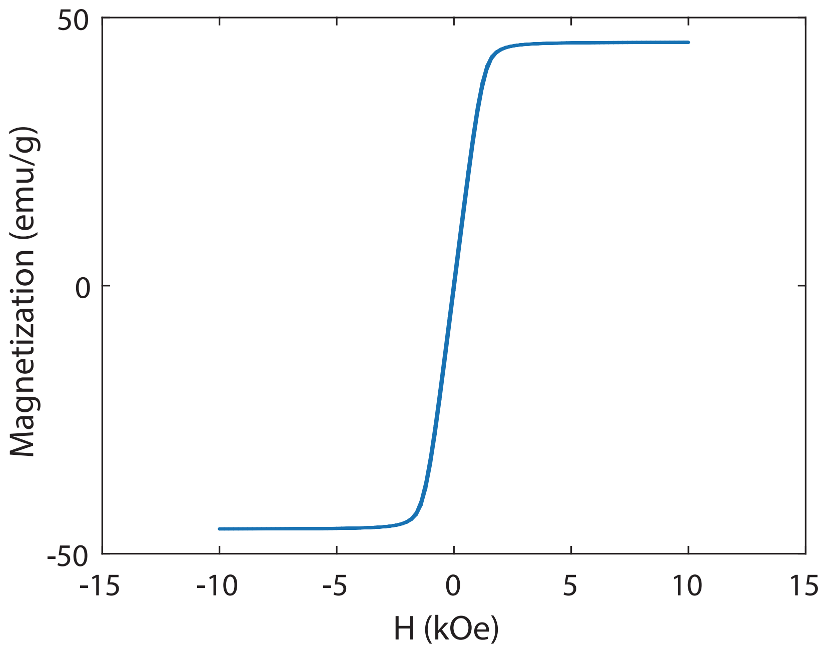

FIG. **Supplementary Fig. 8. Mgantization of YIG at 2 K.**

Supplementary References:

* Email: ustbxuxiaoying@gmail.com, yufanli@cuhk.edu.hk, clchien@jhu.edu

[1] T. L. Francavilla and R. A. Hein. The observation of a transverse voltage at the superconducting transition of thin films. *IEEE Transactions on Magnetics*, 27(2):1039–1042, March 1991.

[2] P. Vašek. Transport in MgB_2 near critical temperature. *physica status solidi c*, 3(9):3096–3099, September 2006.

[3] M.S. da Luz, F.J.H. de Carvalho, C.A.M. dos Santos, C.Y. Shigue, A.J.S. Machado, and R. Ricardo da Silva. Observation of asymmetric transverse voltage in granular high-Tc superconductors. *Physica C: Superconductivity and its Applications*, 419(3):71 – 78, 2005.

[4] A. Segal, M. Karpovski, and A. Gerber. Inhomogeneity and transverse voltage in superconductors. *Phys. Rev. B*, 83(9):094531, March 2011.
